# Disruption of Adipokinetic Hormone Mediated Energy Homeostasis Has Subtle Effects on Physiology, Behavior and Lipid Status During Aging in *Drosophila*

**DOI:** 10.3389/fphys.2018.00949

**Published:** 2018-07-20

**Authors:** Andrea Bednářová, Aleš Tomčala, Michaela Mochanová, Dalibor Kodrík, Natraj Krishnan

**Affiliations:** ^1^Department of Biochemistry, Molecular Biology, Entomology and Plant Pathology, Mississippi State University, Starkville, MS, United States; ^2^Biology Centre, Institute of Entomology, Academy of Sciences, České Budějovice, Czechia; ^3^Biology Centre, Institute of Parasitology, Academy of Sciences, České Budějovice, Czechia; ^4^Faculty of Science, University of South Bohemia, České Budějovice, Czechia

**Keywords:** adipokinetic hormone, aging, AKH signaling, lipid status, senescence, energy homeostasis

## Abstract

The impact of disruption of adipokinetic hormone (AKH) signaling was studied during aging in *Drosophila* in a sexually dimorphic manner. A mutant (*Akh*^1^) producing a non-functional AKH peptide was compared with isogenized wild-type controls (*w*^1118^), and *Akh*-rescue line where AKH was ectopically expressed in the mutant background (*EE-Akh*). Longevity, fecundity, and locomotor activity rhythms remained unaffected by lack of AKH signaling. While the strength of rhythms declined in general with age across all fly lines tested this was more so in case of *Akh*^1^ flies. Negative geotaxis was significantly impaired in *Akh*^1^ flies. Only young *Akh*^1^ flies of both sexes and old *Akh*^1^ females showed significantly higher body weight compared to age-matched iso-control flies (except in case of *EE-Akh*). Expression of genes involved in energy homeostasis and aging indicated that *dTOR* and *Akt* expression were elevated in *Akh*^1^ flies compared to other genotypes, whereas *AMPK* and *dFoxO* expression levels were significantly reduced. Multivariate analysis of the distribution of lipid species revealed a significant accumulation of specific diglyceride (DG) and triglyceride (TG) lipid species, irrespective of sex, attributable in part due to lack of AKH. Moreover, irrespective of lack of AKH, older flies of all genotypes accumulated TGs. Taken together, the results strongly suggest that disruption of AKH has very subtle effects on physiology, behavior and lipid status during aging.

## Introduction

The phenomenon of aging has been characterized as irreversible in all species and is accompanied by a gradual and progressive decline in all physiological functions (López-Otín et al., 2013). This functional decline at all levels of biological organization – molecular, cellular, tissue-level, and organismic – is the hall-mark of aging and several factors have been implicated to be playing a role as either the cause or a consequence of this phenomenon. A major proximal cause of this phenomenon is the progressive decline in cellular homeostatic mechanisms. A crucial requirement for cellular homeostasis is an effective and robust mechanisms for regulation of energy metabolism. Disruptions in the maintenance of energy balance increases the risk of diseases and is detrimental to healthy aging. This begs the question: does impaired energy homeostasis impact/accelerate the process of aging? The fruit fly *Drosophila melanogaster* is an excellent model for investigations on the characterization and elucidation of aging processes and regulatory signaling mechanisms. Thus, we chose to investigate the effects of disruption of energy homeostasis, which is mediated in part by adipokinetic hormone (AKH), in *Drosophila* on some senescence characteristics during aging in a sexually dimorphic manner.

All higher animal organisms including *Drosophila* constantly adapt their energy balance through metabolic regulation of nutrient (sugar, lipid, and amino acid) homeostasis. In insects, this regulation is primarily achieved by AKH [which is a member of the red pigment concentrating hormone/adipokinetic hormone (RPCH/AKH) neuropeptide family] (Gäde et al., 1997). Insect AKHs are analogous to mammalian glucagon in vertebrate body which is mainly involved in the mobilization of energy reserves, mainly glucose and thereby regulates blood glucose levels under physiological as well as stress conditions (Jones et al., 2012; Bednářová et al., 2013). In *Drosophila*, a single *Akh* gene encodes the peptide hormone precursor (79 amino acids), the N-terminal signal peptide (22 amino acids), the active AKH octapeptide (Drome-AKH; pGlu-Leu-Thr-Phe-Ser-Pro-Asp-Thr-NH_2_), as well as the carboxy-terminal AKH associated peptide (49 amino acids) (Schaffer et al., 1990; Noyes et al., 1995). The Drome-AKH is produced and stored in the corpus cardiacum cells (part of the ring gland in the larvae) and released into the hemolymph upon necessity (Kim and Rulifson, 2004; Lee and Park, 2004). The involvement of AMP activated kinase (AMPK) in regulating AKH secretion has been reported in adult *Drosophila* (Braco et al., 2012). Most studies on *Drosophila* model to date have focused on the immediate effects of loss of AKH function on carbohydrate and lipid metabolism and response to starvation (Lee and Park, 2004; Isabel et al., 2005; Bharucha et al., 2008; Baumbach et al., 2014a,b; Sajwan et al., 2015). Recently, it has been demonstrated that AKH signaling is essential also for nutritional and even oxidative stress responses in adults (Bednářová et al., 2015; Gáliková et al., 2015; Zemanová et al., 2016).

In flies, two lipocatabolic systems have been identified so far that influence energy storage, one which is reliant on AKH (Grönke et al., 2007) and the other on the adipose triacylglyceride (TAG) lipase homolog *brummer* (Grönke et al., 2005). Additionally, nutrient-specific signaling pathways such as insulin and TOR (target of rapamycin) signaling are also known to be modulators of longevity (Tatar et al., 2003). Interestingly, the transcription factor FoxO (Forkhead box class O) does not actively participate in dietary yeast manipulated modulation of longevity (Giannakou et al., 2008; Min et al., 2008), whereas other players in the TOR pathway may be required. In the present study, to understand the impact of disruption of energy homeostasis on aging, the transcription levels of AMPK (5′-AMP-activated protein kinase), Akt, TOR, and FoxO, were tested. Importantly, the signaling pathway of AKH in transcriptional response to oxidative stress has been revealed prioviously in *D*. *melanogaster* (Bednářová et al., 2015; Kodrík et al., 2015). Downstream of AKH, the FoxO transcription factors (which belongs to a family of conserved proteins that modulate the cellular response to various stimuli), mediate oxidative stress response signals (Brunet et al., 2004; Furukawa-Hibi et al., 2005; Carter and Brunet, 2007). The c-Jun N-terminal kinases are known to phosphorylate FoxO proteins in response to oxidative stress, causing them to be activated by nuclear translocation. Nuclear translocation coupled activation of FoxO followed by transcriptional regulation of classical antioxidant enzymes such as *manganese superoxide dismutase* and *catalase* gene expression has been implicated in cellular responses to ameliorate oxidative stress (Carter and Brunet, 2007; Glauser and Schlegel, 2007). On the other hand, phosphorylation of FoxO by protein kinase B/phosphoinositide kinase 3 (Akt/PI3), promotes the export of FoxO factors to the cytoplasm from nucleus with an inhibition of FoxO transcriptional regulatory activity. The Akt kinase is activated in response to oxidant injury as well in stress situations and serves as a protective anti-apoptic protein during oxidative stress and toxicity (Ma, 2010). AMPK is another kinase also involved in oxidative stress response. It is typically activated by cellular stress that results in ATP depletion and AMP production (Kahn et al., 2005; Fuentes et al., 2013). The other transcriptional factors, TOR proteins (members of the phosphatidylinositol kinase-related kinase family) are also one of the players in FOXO-Sestrin-AMPK-TOR pathway in *Drosophila* (Bednářová et al., 2015).

Given that with aberrant AKH signaling, no overt effects are observed under non-stressed conditions, we hypothesized that lack of AKH may have an impact on senescence characteristics during the process of aging since disruptions in energy homeostasis are likely to impact the process of healthy aging. To test this hypothesis we utilized the *Akh* loss of function mutant generated using engineered nucleases by means of a specific TALEN pair (Sajwan et al., 2015). This *Akh*^1^ mutant has a 3 bp deletion leading to the loss of leucine at position 2 of the octapeptide with the remaining carboxy-terminal associated peptide intact. In parallel, we used flies with ectopic expression of AKH in mutant *Akh*^1^ background (*EE-Akh*) using the UAS-Gal4 system (Sajwan et al., 2015). The lipid status (both structural and storage) was analyzed by HPLC-ESI MS and a comparison was made among all fly lines used in this study which included age (young vs. old) as well as sex differences. Thus, our objective was to investigate the effects of loss of AKH function and the consequent disruption of energy homeostasis on aging characteristics during adult life in *Drosophila*.

## Materials and Methods

### *Drosophila* Stocks and Maintenance of Cultures

Cultures of *D. melanogaster* were maintained on a diet of agar (1%), cornmeal (6.25%), molasses (6.25%), and active dry yeast (Red Star, 6.25%) at 25°C in 12 h Light-12 h Dark cycles (with an illumination intensity of ∼2,000 lux). Fly lines used in this study were the *Akh* mutant *Akh*^1^ generated by a 3 bp deletion causing a loss of the second aa in the AKH octapeptide, with the remaining carboxy-terminal AKH-associated peptide unaffected. These flies were created using the transcriptional activator like effector nucleases (TALEN) described in our previous study (Sajwan et al., 2015). Rescue flies carrying the construct to restore AKH function in the *Akh*^1^ mutant was generated by ectopic expression of transgenic *UAS-Akh*^+^. In this case, the original *Akh*^1^ mutant was combined with the *Act-Gal4* driver and UAS-*Akh* transgene by crossing *w*^1118^; *Act-Gal4*/*CyO*, *Act-GFP*; *Akh*^1^ flies to *w*^1118^; *UAS-Akh*^+^; *Akh*^1^ ones. These flies were designated as (ectopically expressing *Akh*) *EE-Akh*. The *Akh-*expressing flies were selected (*w*^1118^; *Act-Gal4*/*UAS-Akh*^+^; *Akh*^1^) based on the absence of green fluorescence (or the *Cy* marker in adults) among the F_1_ progeny (Sajwan et al., 2015). Backcrossing of mutants and transgenes to the *w*^1118^ background was conducted for six to eight generations for isogenization. Female and male flies were divided into three different age groups: young flies ∼5 days old, middle age flies ∼35 days old, old flies ∼50 days old. A part of the experimental work also utilized CRISPR/Cas9 mutants of AKH. These mutants were a kind gift from the laboratory of Dr. Kuhnlein (Gáliková et al., 2015). Details on generation of Akh^*A*^, Akh^*AP*^, Akh^*SAP*^, Akh^*R*^ and their genetically matched controls have been reported previously (Gáliková et al., 2015).

### Lifespan Measurements

Lifespan measurements were conducted in three cohorts (replicates) of ∼80 mated males and 80 mated female flies of each genotype (*n* = 240) separately. These flies of a specific sex and genotype were kept separately in round bottom polypropylene bottles (8 oz, Genesee Scientific, San Diego, CA, United States) inverted over 60 mm Falcon Primaria tissue culture dishes serving as lids (Becton Dickinson Labware, Franklin Lakes, NJ, United States) containing diet (15 mL). The diet dishes were replaced on alternate days after tapping flies down to the bottom of the bottle. Mortality was scored daily, and Kaplan–Meier survival curves were generated and Log-rank (Mantel–Cox) tests were conducted using GraphPad Prism (v 5.01). The Gompertz–Makeham maximum likelihood estimates (MLE) were conducted using WinModest (v.1.0.2) (Krishnan et al., 2009).

### Circadian Locomotor Activity Analysis

Entrainment of flies of each sex, genotype, and age group was conducted in 12:12 LD cycle at 25°C for 3 days for acclimatization to the Trikinetics *Drosophila* locomotor activity monitor (Waltham, MA, United States). The locomotor activity of young (3–5 days), middle aged (30–35 days), and old (45–50 days) male and female flies of each genotype was recorded for 3 days in LD 12:12, followed by 10 days in constant darkness (DD) (Pfeiffenberger et al., 2010). Locomotor activity counts (actograms) was recorded by the number of infrared beam crossings of the individual flies collected in 15 min bins. The total number of beam crossings in LD cycles was averaged over a period of 3 days in LD for all flies of a particular sex and genotype for generating the average daily activity profiles. Fast Fourier Transform (FFT) analysis of activity data during DD was conducted using CLOCKLAB software (Actimetrics; Coulbourn Instruments, Whitehall Township, PA, United States) as a quantitative measure of circadian rhythmicity. Flies with FFT values <0.04 were classified as arrhythmic, flies with FFT values ranging from 0.04 to 0.08 were categorized as weakly rhythmic, whereas flies with FFT > 0.08 were considered strongly rhythmic. Flies which showed a single peak in the periodogram (both with weak and strong rhythms) were included for calculations of free-running period using the CLOCKLAB software.

### Rapid Iterative Negative Geotaxis Assay (RING)

The RING assay was used to test the negative geotaxis response for males and females of each genotype at room temperature (25 ± 1°C) (Gargano et al., 2005). Twenty five male and female flies (independently) in three replicates for each age group (5, 35, and 50 days) and of each genotype were used in the RING assay. After a 3 min acclimatization period in the RING apparatus, the apparatus was rapped sharply (3×) to generate a negative geotaxis response. The climbing movement of the flies in tubes was recorded as a movie and digital images captured for a duration of 4 s after initiating the behavior. This was subsequently used for data analyses. This process was repeated five times (interspersed with ∼30 s rest between trials). Trials were also randomized to ensure genotypes had sufficient recovery time. The climbing performance (average height climbed by all flies during 4 s) was averaged for three vials of a given genotype and given age group and represented graphically.

### Body Weight Measurement

To measure the body weight, a total of 100 flies from each genotype, sex and age group were weighed using Mettler Toledo AL54 precision balance. Flies were weighed in groups of 10 individuals and 10-independent measures were taken, so a total of 100 individuals were weighed for each sex, genotype, and age group and was used for subsequent statistical analysis.

### Fecundity Assay

Fecundity was measured as number of eggs laid daily by individual females during the first 10 days following mating. Six virgin females and six males of the same genotype were placed on a standard diet with no addition of yeast to avoid the possibility that the fecundity can be increased by the use of yeast. Flies were put on a fresh diet daily and the number of eggs laid counted under a stereo-microscope. The average number of eggs laid by a single female (during the first 10 days of their life) was recorded and represented as fecundity. Care was taken to exclude females that died or escaped during the experiment from the analyses.

### HPLC ESI MS of Lipids

Lipids from whole animals were extracted following homogenization with chloroform: methanol (2:1) solution (Folch et al., 1957; Koštál and Šimek, 1998). Aliquots of 100 μl were processed by HPLC (Accela, Thermo Fisher Scientific, San Jose, CA, United States) ESI MS (electrospray ionization-high mass spectrometry using LTQ-XL mass spectrometer, Thermo Fisher Scientific, San Jose, CA, United States) for lipid determinatiion. The identity of particular lipid species was determined based on ionization behavior and fragmentation patterns and confirmed by HRMS (high resolution mass spectrometry using Orbitrap Q-Exactive Plus with Dionex Ultimate 3000 XRS pump and Dionex 3000 XRS Open autosampler, Thermo Fisher Scientific, San Jose, CA, United States). The detailed methods and instruments settings were as described previously (Tomčala et al., 2017). The software Xcalibur (v 2.1, Thermo Fisher Scientific, San Jose, CA, United States) was used for acquiring and processing data from both instruments. Phosphatidylcholine 17:0/17:0 was used as an internal standard.

### Multivarate Analysis of Lipids

The data obtained from the areas of TGs, DGs, and structural lipids peak areas were subjected to statistical analysis using ordination methods: detrended correspondence analysis (DCA) – for all data; principal component analysis (PCA), redundancy analysis (RDA), Monte-Carlo permutation test (unrestricted permutations, *n* = 999) – for linear data. The data was transformed by using internal standard peak area and weight of particular sample. In the canonical analysis (RDA) were the age, sex, and genotype as a categorical predictor. Monte-Carlo permutation test were used for determining statistical significance. DCA, PCA, RDA, and Monte-Carlo permutation test analyses was conducted using the Statistics software CANOCO 4.5 (Biometris, Plant Research International, Wageningen University & Research, Netherlands).

### Quantitative Real-Time Polymerase Chain Reaction

The mRNA expression of *AMPK*, *dTOR*, *Akt*, and *dFoxO* genes was measured in three independent bioreplicates of female and male flies (5, 35, and 50 days old) of each genotype. Tri Reagent (Sigma, St. Louis, MO, United States) was used for extraction of total RNA from 25 flies of each genotype and sex. Takara Recombinant DNase I (Clontech Laboratories, Inc., Mountain View, CA, United States) was used to remove genomic DNA from samples. cDNA synthesis was conducted using the iScript cDNA synthesis kit (BioRad, Hercules, CA, United States). Quantitative real-time PCR (qRT-PCR) was conducted under default thermal cycling conditions, with a dissociation curve step on the Eppendorf realplex^2^ Mastercycler (Eppendorf, United States). Each reaction mixture contained Power SYBR Green (Applied Biosystems), 10 ng cDNA, and 400 nM primers. Primer sequences have been listed in Supplementary Table S1. Relative expression levels were analyzed using the 2^−ΔΔCT^ method (Livak and Schmittgen, 2001) with mRNA levels normalized to the gene *rp49*. Relative mRNA amplitude was calculated with respect to young (5 days old) male *w*^1118^ flies whose expression for a particular gene was set at 1.

### Statistical Analysis

Statistical analysis of all parameters except longevity was conducted using one way ANOVA (RING and body mass data) with Tukey’s *post hoc* tests or two-way ANOVA (Locomotor activity and gene expression) with Bonferroni post-test (ezANOVA v 0.98). For longevity data, the Log-Rank (Mantel–Cox) test was used to analyze the Kaplan–Meier survival curves. Statistical significance was set at *p* < 0.05. Graphs were generated using GraphPad Prism v 5.01 (GraphPad Software, Inc., San Diego, CA, United States).

## Results

### Lack of AKH Does Not Compromise Lifespan

**FIGURE 1:**
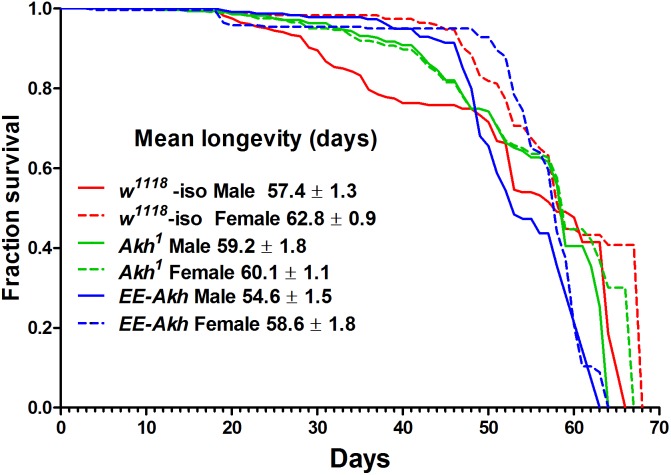
Kaplan–Meier survival curves of *w*^1118^-iso, *Akh*^1^, and *EE-Akh* male and female flies under 12 h light:dark (LD) cycles and *ad libitum* feeding conditions. Log-rank (Mantel–Cox) test revealed significant differences (*p* < 0.0014) between survival curves of male vs. female flies of isogenized controls and *EE-Akh* flies but not between males and females of *Akh*^1^ flies. Also no difference in survival curves between males of isogenized controls vs. *Akh*^1^ flies was recorded. Females were, however, significantly different in their survival curves among genotypes. Survival data has been depicted as mean survival values ±SD. Data was obtained from three independent replicates of 80 flies of each sex for each genotype (*n* = 240).

We conducted longevity assays to determine if disruption of AKH signaling and the consequent disruption of energy homeostasis (primarily lipid mobilization) has an impact on longevity of flies. The assessment for lifespan using Kaplan–Meier survival curves indicated some sex-specific and genotypic differences (**Figure 1**). There was a marginal but non-significant increase in mean longevity in *Akh*^1^ male mutants (59.2 ± 1.8 days) compared to *w*^1118^ controls (57.4 ± 1.3 days) and this was the highest recorded longevity among males of all genotypes (**Figure 1**). On the contrary, the *Akh*^1^ female mutants longevity was significantly decreased (60.1 ± 1.1 days, *p* < 0.001) compared to that of *w*^1118^ controls (62.8 ± 0.9 days) (**Figure 1**).

Upon comparing males and females of a particular genotype, sex specific differences in the mean longevity was observed (**Figure 1**). However, no significant difference in longevity was found between *Akh*^1^ males (59.2 ± 1.8) and females (60.1 ± 1.1). The mortality parameters reflected the male- or female-specific mortality hazards derived from the Gompertz–Makeham model and MLE (Supplementary Table S2). Mortality hazards over the period of aging were not significantly different in *Akh*^1^ flies [Ln mortality (μ_x_) at day 14 = −5.07 ± 0.001 and at day 54 = −2.27 ± 0.01] when compared to their isogenized controls (μ_x_ at day 14 = −5.06 ± 0.001, at day 54 = −2.26 ± 0.01) or those with *Akh* function restored (μ_x_ = −5.07 ± 0.001, at day 54 = −2.26 ± 0.01) irrespective of sex. Thus, under unstressed-laboratory conditions, lack of AKH signaling does not compromise lifespan.

### Age-Related Senescence in Locomotor Activity Is Mildly Affected by Lack of AKH

**FIGURE 2:**
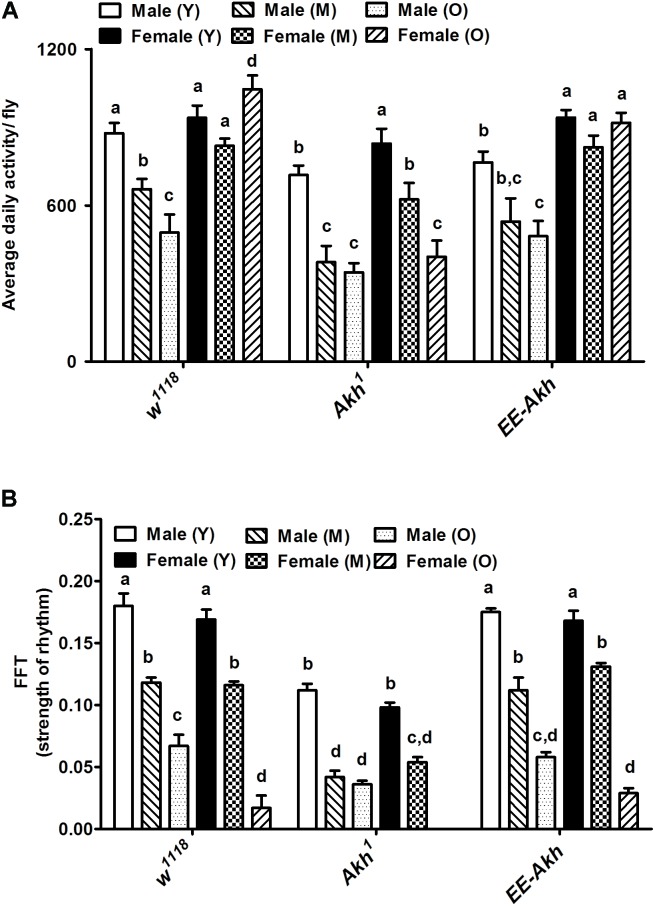
Graphical representation of **(A)** the average daily activity per fly, and **(B)** strength of the rhythm exhibited by rhythmic flies. Average daily activity was computed as beam crossings in 15 min bins over 3 days of LD cycles (see section “Materials and Methods” for more details). Comparisons for various parameters were made between male (young vs. middle age vs. old), female (young vs. middle age vs. old) and young (male vs. female), middle (male vs. female), and old (male vs. female) for each genotype as well as among genotypes. Bars with different superscripts (in small alphabets) are significantly different at *p* < 0.05 (two-way ANOVA with Bonferroni multiple comparisons test). Comparisons as were made across all combinations, within a genotype (sex and age) as well as among genotypes. Data are represented mean ± SEM (*n* = 32 flies of each sex, genotype, and age).

Since AKH is involved both in locomotor activity stimulation and in nutrient mobilization, we decided to check if disruption of AKH has an impact on circadian locomotor activity during aging. The average daily activity in males declined with age across all genotypes (**Figure 2A**). However, this was quite the opposite in females with old flies exhibiting either comparable activity levels or even elevated activity compared to young except in case of *Akh*^1^ mutants. Young *Akh*^1^ and *EE-Akh* males showed the lesser activity compared to isogenized controls. Old females of isogenized control and *EE-Akh* flies showed higher average daily activity compared to age-matched-males while it was not significantly different in case of *Akh*^1^ mutants (**Figure 2A**).

Age-associated decline in strength of rhythms was recorded among both males and females across genotypes (**Figure 2B**). Interestingly, rhythm strength was the least in young male *Akh*^1^ mutants when compared to males of the other two genotypes of similar age. The same pattern was also observed among females with *Akh*^1^ mutants exhibiting the least strength of rhythm (**Figure 2B**).

Circadian locomotor activity rhythms changed significantly across all genotypes with age (young, middle, and old) in both sexes (Supplementary Table S3). Male *w*^1118^ flies exhibited decline in rhythmicity from 100% in young to 20% in old. In females, only 18% of old flies remained rhythmic. In *Akh*^1^ mutants with no functional AKH, a decline in rhythmicity in males was recorded from 95% in young to 24% in old; however, in old females, no rhythmic individuals (0%) were recorded. The rhythmicity of young *EE-Akh* was lower, 75% of control ones and thus its decrease in old ones was less marked (31%). Similarly, in *EE*-*Akh* females, rhythmicity was 72% and 17% in young and old ones, respectively (Supplementary Table S3). Thus, subtle differences were recorded in activity levels and strength of rhythm as a result of disruption of AKH signaling during aging.

### Disruption of AKH Signaling Impairs Negative Geotaxis Response in an Age-Dependent Manner

**FIGURE 3:**
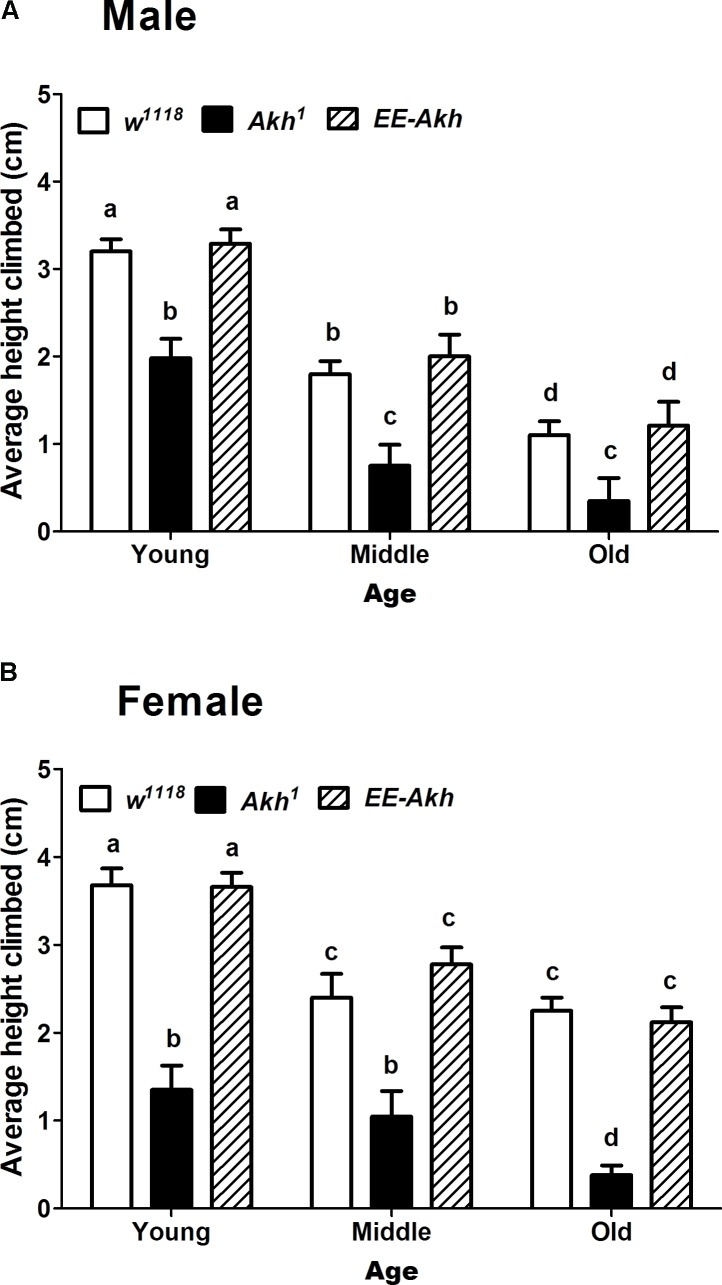
Rapid iterative negative geotaxis (RING) assay of experimental (young∼3–5 day old, middle ∼35 day old, and old ∼50 day old) **(A)** male and **(B)** female flies of various genotypes. Bar graphs represent average height climbed by each genotype. Data are presented as mean ± SD (*n* = 75 flies of each genotype sex and age). Data were analyzed by two-way ANOVA with genotype and age being fixed effects, followed by Bonferroni *post hoc* test. Comparisons were made across all combinations, among genotypes within a specific age and also among genotypes across different ages. Bars with different superscripts (small alphabets) are significantly different at *p* < 0.05.

The negative geotaxis response is a measure of robustness or healthspan during the process of aging. Thus, we checked if the negative geotaxis response is affected in flies with disrupted AKH signaling. In general the females exhibited more negative geotactic response compared to males (**Figures 3A,B**). Interestingly *Akh*^1^ mutants (both males and females) exhibited significantly less climbing ability compared to the other two genotypes at all ages examined. The climbing ability showed a marked decline with age across genotypes and this was more so for the *Akh*^1^ mutants compared to *w*^1118^ controls for both males and females (males: 5.6 times for *Akh*^1^ vs. 2.9 times for *w*^1118^, females: 3.5 times for *Akh*^1^ vs. 1.6 times for *w*^1118^) (**Figures 3A,B**). Thus, lack of AKH has a significant impact on negative geotaxis response in young age and progressively deteriorates in an age-dependent manner.

### Lack of AKH Impacts Body Weight Only in Young Flies of Both Sexes and Old Female Flies

**FIGURE 4:**
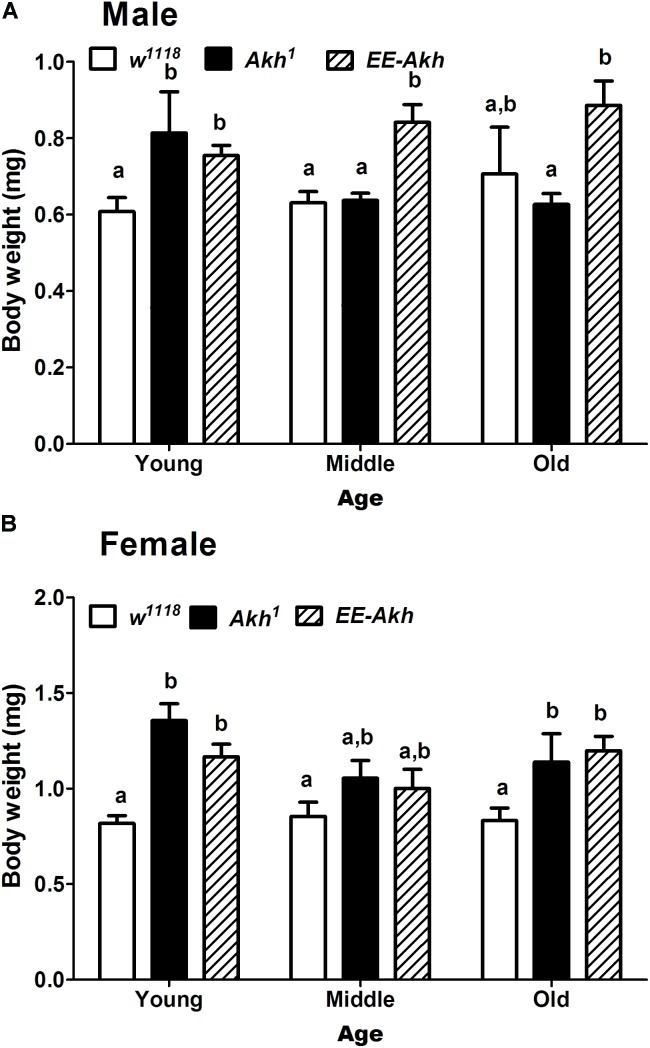
Body weight of experimental (young∼3–5 day old, middle ∼35 day old, and old ∼50 day old) **(A)** male and **(B)** female flies of various genotypes. Data are presented as mean ± SD (*n* = 100 flies of each genotype, sex, and age group). Data were analyzed by two-way ANOVA with genotype and age being fixed effects followed by Bonferroni’s *post hoc* test. Comparisons were made across all combinations, among genotypes within a specific age and also among genotypes across different ages. Bars with different superscripts (small alphabets) are significantly different at *p* < 0.05.

The primary role of AKH is in mobilizing energy reserves in the form of lipids to generate fuel for locomotion and other activities demanding energy. The lack of AKH has been correlated to starvation resistance because of enhanced levels of lipid reserves which are under-utilized, thus providing fuel for energy requiring processes during conditions of starvation. Lack of AKH thus would suggest an obese phenotype due to lack of mobilization of lipids. Thus, we checked if body weight is affected during the process of aging in all fly lines. No significant differences were observed with age in body weight among males of isogenized control. The body weight of young *Akh*^1^ males was significantly higher than that in controls. Also, middle and old *EE-Akh* flies’ body weight was the highest of those among all genotypes and ages. Interestingly, females of *Akh*^1^ and *EE-Akh* mutants at young and old age did show significant increase in body weight compared to isogenized control (**Figures 4A,B**). In general, females showed higher body weight compared to males of a genotype (**Figures 4A,B**). Thus, lack of AKH impacts only young flies (both sex) and old female individuals with an obese phenotype but this is not the case during aging.

### Lack of AKH Signaling Does Not Impact Fecundity

**FIGURE 5:**
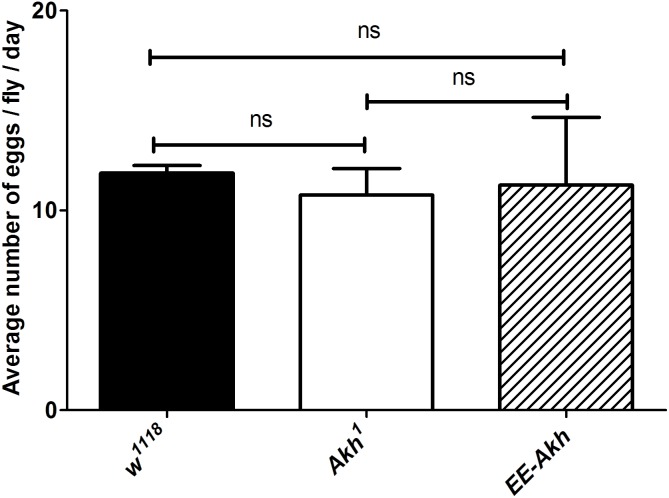
Mean number of eggs laid per day per female fly. Data are represented as mean ± SD of mean (*n* = 18 flies of each genotype). The average fecundity among the genotypes was not significantly different as analyzed by one-way ANOVA (Kruskal–Wallis test, *p*-value 0.808) with Dunn’s multiple comparison test.

A decline in fecundity precedes the manifestation of reproductive senescence. Fecundity is tightly related to storage of lipid reserves for the developing eggs as well as reproductive maturation and ovisposition are energy requiring processes. Thus, we investigated if disruption of AKH signaling could impact fecundity of young female flies. No significant difference in fecundity was recorded among *Akh*^1^ females compared to control or AKH function rescued flies (*EE-Akh* flies) (**Figure 5**). Hence, lack of AKH does not affect fecundity.

### Disruption of AKH Signaling Has a Differential Effect on Expression of Genes Involved in Energy Sensing and Aging

**FIGURE 6:**
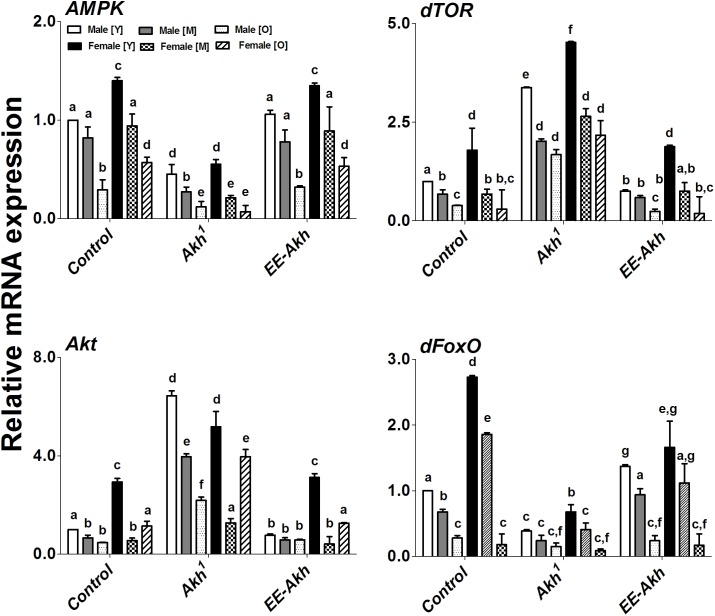
Gene (qPCR) expression of key signaling members involved in energy sensing and aging. Expression levels of *AMPK, dTOR, Akt*, and *dFoxO*, genes in males and females at young, middle, and old ages of w^1118^- isogenized controls, *Akh*^1^ and *EE-Akh* flies. Relative mRNA expression of control was set as a reference (=1). Values represent mean ± SEM of three independent bio-replicates (*n* = 75 flies of each genotype sex and age). Two-way ANOVA with Bonferroni *post hoc* multiple comparisons test was conducted to separate out the means. Comparisons as were made across all combinations, within a genotype (sex and age) as well as among genotypes. Bars with different superscripts (small alphabets) are significantly different at *p* < 0.05.

Improved healthspan coupled with extended lifespan reflect a finely tuned regulation of metabolic energy homeostasis which is associated robust cellular housekeeping with enhanced resistance to stress. Thus, we investigated if transcription factors and genes involved in all these processes are affected by lack of AKH signaling during the process of aging. The expression of *AMPK*, *dTOR*, *Akt*, and *dFoxO* varied significantly among genotypes, sex as well as with age. In case of the energy sensor *AMPK*, *Akh*^1^ mutant males and females showed the least expression compared to the other two genotypes while in general females of young age showed significantly higher expression of *AMPK* compared to age matched male flies within a genotype (except in case of *Akh*^1^ flies where the expression levels were similar) (**Figure 6**). Control females along with *EE-Akh* females had similar levels of *AMPK* expression at each specific age. A gradual age-related decline in *AMPK* expression was observed across both sexes of all genotypes. A similar pattern was observed for *dFoxO* expression.

Interestingly *dTOR* expression was the highest in *Akh*^1^ mutants compared to all other genotypes across both sexes and all ages (**Figure 6**). An age associated decline in *dTOR* expression was observed across genotypes, however, in certain instances there was no significant difference between middle and old flies in either males or females across genotypes. While a similar pattern was recorded for *Akt*, it was observed that in old females of all genotypes, there was a significantly higher expression of *Akt* compared to middle ages flies in both sexes across genotypes (**Figure 6**).

In general, young females showed higher expression of genes studied compared to age matched males across genotypes but this did not hold true with other ages (**Figure 6**). Taken together, our results do suggest that lack of AKH signaling does impact the expression of genes and transcription factors which are involved in energy homeostasis and stress resistance in a sexually dimorphic manner.

### Lack of AKH Has a Mild Effect on the Status of Lipid Species Irrespective of Sex or Age

**FIGURE 7:**
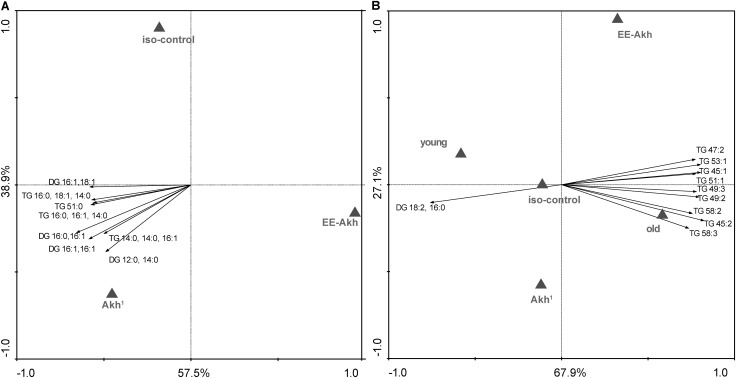
Multivariate analysis of lipid status in different genotypes. **(A)** RDA diagram of lipidomic data, obtained via HPLC ESI MS, of iso-control (*w*^1118^), *Akh*^1^, and *EE-Akh* fly lines. The multivariate analysis showed differences among depicted fly lines at *p* < 0.05. The main lipid species responsible for data separation are depicted in the diagram. **(B)** RDA diagram of lipidomic data, obtained via HPLC ESI MS, of iso-control (*w*^1118^), *Akh*^1^, and EE-Akh fly lines in young and old flies. The multivariate analysis showed differences among depicted fly lines at *p* < 0.01. The main responsible lipid species for data separation are depicted in the diagram. Both diagrams depict the graphical result of RDA (a canonical version of PCA). The labels of x and y-axis in percentage represent the variability in the dataset as described in the diagram. Triangles represent particular fly lines and the arrows represent lipids. The direction of the arrows indicates a higher occurrence of the lipid species or its presence (pointing to triangle) or absence (pointing to opposite side) in a defined fly line. The length of the arrow represents the statistical power for presence of a particular lipid species.

Given that AKH is involved in the mobilization of lipids to fuel energy demanding processes, we investigated if disruption of AKH signaling could impact the lipid status during the process of aging. This study was conducted only in young vs. old individuals of all genotypes studied in a sex specific manner to obtain a snap-shot of what lipid species or lipid classes are affected (Supplementary Table S4). A multivariate redundancy analysis (RDA) of lipid species among the different fly lines revealed no significant differences among structural lipids (Supplementary Figure S1). However, storage lipids such as diglycerides (DG) and triglycerides (TG) did vary significantly among the genotypes tested (**Figure 7A**). Flies with disrupted AKH signaling had a tendency to accumulate specific species of DGs and TGs (**Figure 7A**). Interestingly when age (young vs. old) was taken into consideration, we found that lack of AKH has only very minor effect on the lipid status whereas old flies of all genotypes in general showed an abundance of TGs (**Figure 7B**). Thus, there is an accumulation of fatty acids with odd-numbered carbons (hypertriglyceridemia) during the process of aging irrespective of presence or lack of AKH. The sex of the flies does not seem to affect the lipid status across genotypes studied.

Interestingly, when we tested flies with mutation in *Akh* gene generated using CRISPR technique, we obtained similar results for overall structural lipids which did not vary with the type of mutation (in AKH-hormone – AKH^*A*^, or just the propeptide – AKH^*AP*^, or both hormone and propeptide- AKH^*SAP*^) compared to isogenized controls (Supplementary Figure S2). On the other hand, AKH^*SAP*^ flies significantly differed from age-matched controls and other mutants with lower TGs, whereas AKH^*AP*^ fly lines revealed the highest amount of TGs (Supplementary Figure S3). When age was taken as a factor (young vs. old), in contrast to our fly lines created using TALENS, the CRISPR mutants at the young age showed enhanced accumulation of TGs, whereas older individuals did show accumulation of two TG species (45:3 and 45:2) (Supplementary Figure S4). Again, as in case of our fly lines, in the CRISPR mutants, no effect of sex was observed on differential accumulation of lipids.

Taken together, our results suggest that lack of AKH signaling has relatively mild effects on the lipid status during the process of aging, irrespective of sex.

## Discussion

In general, aging is characterized by a widespread loss of function at all levels of biological organization: molecular, cellular, tissue, and organismal (Greer and Brunet, 2008; Murphy and Partridge, 2008; Rando and Chang, 2012). Since the process of aging is strongly linked to maintenance of homeostasis, we targeted the effects of aberrant energy homeostasis on senescence characteristics during aging in *Drosophila*. Therefore, we focused on physiological and behavioral attributes such as longevity, daily locomotor activity, strength of locomotor activity rhythms, negative geotactic response, body weight changes with age and reproductive output (fecundity) as read-outs of senescence characteristics in a sexually dimorphic manner. Additionally, we also targeted the expression of specific genes involved in energy sensing and aging processes – *AMPK*, *dTOR*, *Akt*, and *dFoxO.*

The outcomes of our study indicate a certain role of AKH in the process of senescence, manifested in a sexually dimorphic manner. Under natural/non-stressed conditions *Akh*^1^ flies exhibited slightly enhanced longevity compared to isogenized controls or flies with restored AKH function. On the other hand, *Akh*^1^ flies have been demonstrated to have higher resistance to starvation (Sajwan et al., 2015; Zemanová et al., 2016). Similarly, AkhR mutants (flies mutated in AKH receptor) were also reported to display higher resistance to starvation (Lee and Park, 2004; Caers et al., 2012; Gáliková et al., 2015). Hence, under the normal conditions lack of AKH does not appear to impact longevity in a negative manner.

Next, we examined if locomotor activity rhythms are disrupted by lack of AKH or if rhythms change with age. Lack of functional AKH did not have any effect on locomotor activity rhythms as shown previously (Lee and Park, 2004), however, this study showed that the females of *Akh*^1^ showed arrhythmicity during old age. This testifies that AKH signaling has to be finely regulated to maintain proper locomotor activity rhythms in old flies, a lack of functional AKH will result in arrhythmic behavior. However, this was not observed in case of male flies. This points to a sexually dimorphic role for AKH in the process of controlling locomotor activity rhythms. Moreover, the strength of locomotor activity was directly related to lack or presence of functional AKH. Lack of functional AKH resulted in weaker rhythm while presence of AKH signaling resulted in stronger rhythms. While it has been established previously that circadian locomotor rhythmicity goes down with age (Umezaki et al., 2012), we found that this behavioral pattern was more prominent in flies lacking AKH.

Negative geotaxis was also linked to AKH signaling. Non-functional AKH impaired the negative geotaxis of flies of both sexes. Since negative geotaxis is a functional readout of aging and senescence, it can be concluded that AKH does impact senescence characteristics in aging *Drosophila*. This result has, however, to be interpreted with caution: it could also be that negative geotaxis, an energy requiring process can be significantly affected in *Akh*^1^ flies as demonstrated by the fact that young flies were significantly slower in their response than age-matched controls. In such case, the impaired negative geotaxis ability may simply be a behavioral characteristic of disrupted AKH signaling rather than health-related.

Interestingly, lack of functional AKH did not result in an overall obese phenotype during older ages but only in young flies. This indicated that body weight/ body mass is not strongly linked to AKH signaling, there may be other players such as insulin which may be linked to not just body weight and homeostasis but also the phenomenon of aging (Broughton et al., 2005; Grönke et al., 2010). Genetic ablation of insulin producing cells during early larval stages has been shown to delay development with an elevation of sugar levels in larval hemolymph (Rulifson, 2002), however, such ablation in adult stage has been shown to prolong lifespan while reducing fecundity with an increase in storage of triglycerides and sugars coupled with enhanced resistance to starvation and oxidative stress (Broughton et al., 2005). It was reported earlier that *Akh*^1^ flies were slightly heavier at young age, suggesting that increased body weight also occurred due to difficulties with nutrient mobilization and metabolic transport regulated by AKH (Sajwan et al., 2015). Higher amounts of stored carbohydrates and lipids have also been reported in flies with RNAi-depleted AKH and AkhR mutants (Bharucha et al., 2008; Baumbach et al., 2014a,b) most likely for similar reasons. The fact that the rescue construct *EE-Akh* also showed similar phenotype as *Akh*^1^ flies in the present study could be partly because of some form of hybrid vigor since these flies were generated by crossing a Gal-4 line to a UAS line. Mutations in *Akh* have been reported to result in obesity (Giannakou et al., 2008). However, this phenotype is from the adulthood-specific requirement of AKH signaling rather than any developmental defect (Gáliková et al., 2017). The obese phenotype of the Akh^*A*^ and AkhR^1^ loss-of-function mutants can be recapitulated by RNAi against the AKH receptor AkhR (AkhRi) driven specifically during adulthood by using the ubiquitously expressed daughterless-GeneSwitch-Gal4 (da-GS) (Gáliková et al., 2017). This anti-obesity effect of AKH signaling is localized in the fat body, since it was demonstrated that the fat body specific AkhRi driven by the FBI-26-GeneSwitch-Gal4 (FBI- 26-GS) triggers obesity. On the other hand, it was also reported that an increase of AKH signaling by over-expressing the wild-type Akh (either ubiquitously or specifically in fat body) in the adult flies results in reduction of lipid levels (Gáliková et al., 2017). In our study too, the structural lipid levels were unaffected by manipulations of AKH signaling whereas storage lipids were significantly impacted by such manipulations as well as age. Our previous study showed a decrease in metabolic rate associated with AKH deficiency, this suggests a plausible contributing factor to AKH deficiency-induced obesity (Sajwan et al., 2015). Despite this, during aging this phenotype did not hold true in case of males and the reasons for this are unclear.

In insects, certain morphological, physiological, and behavioral traits are subject to natural selection and hence are optimized to enable successful continuation of the species by reproduction. We sought in our work to answer the question if disruption of AKH will have any effect on fecundity. It has been reported earlier that *Drosophila* AKH signaling is dispensable for ontogenesis, locomotion and oogenesis until up to the end of metamorphosis (Gáliková et al., 2015). We also did not observe any significant changes among flies where the AKH production was inhibited compared to controls. Previously AKH was suggested to play a permissive role in reproductive events, primarily as inhibitor of vitellogenin synthesis (Carlisle and Loughton, 1986; Moshitzky and Applebaum, 1990) and also as an activator of catabolic reactions. However, AKH should not be regarded as an “inhibitor of reproduction” in *Drosophila*. AKH is very likely one of the hormones that trigger the mobilization of energetic substrates that are incorporated into the growing oocytes, at least in some insects (Lorenz and Gäde, 2009). Since the formation of energy stores in the fat body coincides with oogenesis in *Gryllus bimaculatus* (Lorenz and Anand, 2004), it is assumed that endocrine regulation of anabolic and catabolic reactions has to be carefully orchestrated. Thus, in crickets, during phases of low physical activity (morning until early afternoon) anabolic reactions occur and are associated with relatively low titers of lipids and AKH in the hemolymph. In the evening and early night, increased locomotor activity is associated with increases in lipid titers in the hemolymph. This is possibly due to an increased release of AKH, which inhibits anabolic reactions while triggering the release of energetic substrates from the fat body. These substrates could then be utilized to fuel flight activity or for oogenesis. In the nematode *Caenorhabditis elegans*, the involvement of an AKH-like peptide in reproductive processes has been demonstrated – when the expression of the receptor or its ligand Ce-AKH/GnRH (an endogenous peptide belonging to the AKH-family), was blocked by RNA-interference, oviposition was reduced by ∼60% compared to control animals (Lindemans et al., 2009). Thus, involvement of AKH in reproductive output seems to be species specific.

An important requirement for metabolic homeostasis involves inter-organ communication whereby organs can coordinate their activities by secreting humoral factors or via nerve impulses. While this is a well-established phenomenon in mammals, not much is known about how such communication occurs in insects. Some studies on *Drosophila* suggest that such “communication” also occurs among their organs through various signaling molecules (Rajan and Perrimon, 2011). A case to point is *dFoxO*. Overexpression of *dFoxO* in flight muscles reduces feeding behavior (Demontis and Perrimon, 2010). Thus, we studied the genes involved in energy sensing process as well as aging. AMPK functions as an energy sensor within the cell and helps maintain energy homeostasis. Thus, AMPK serves a crucial function in regulating energy balance. Lack of AKH signaling significantly downregulated *AMPK* gene expression. This implicates that AKH is crucial for AMPK signaling and vice versa (Braco et al., 2012). The regulation of energy metabolism is a key requirement for cellular homeostasis. Disturbances in the maintenance of energy balance provoke diseases and can jeopardize healthy aging. Since our experiments were conducted under “ideal” conditions and the flies lacking functional AKH were non-stressed, so the real role of AKH was not apparent and hence, a stress paradigm would be required to actually discern the effects of AKH. Additionally, the flies in our study were fed standard diet under *ad libitum* conditions. It is interesting to speculate if manipulations of components of the diet could have revealed an as yet unreported phenotype in *Akh*^1^ mutants.

AMP activated kinase induces stimulation of FoxO which regulates several important cellular functions such as glucose and lipid metabolism, resistance to stress, apoptosis, cell cycle, and also inflammatory responses (Greer and Brunet, 2008; Nakae et al., 2008; Peng, 2008). FoxO factors are important players in longevity via the nuclear factor kappa B (Lin et al., 2004). Indeed, with upregulation of AMPK in the *EE*-*Akh*, we observed a consequent elevation of dFoxO. This was with a concomitant down-regulation of the negative regulators the target of rapamycin (dTOR) and protein kinase b (Akt). It is also known that dTOR negatively affects d*Thor* which is a 4eBP binding protein. In case of lack of functional AKH, a marked up-regulation of the negative regulators of AMPK and dFoxO were observed earlier (Gáliková et al., 2017). A marginal increase (albeit not significant) in expression levels of *Thor* in Akh^*A*^ mutants has been reported, which suggests that signaling by peripheral insulin can be decreased as a consequence of lack of AKH (Gáliková et al., 2017). Interestingly, contrary to our findings in this study, it has been reported previously that trehalose-dependent TOR activation requires AKH (Kim and Neufeld, 2015). However, this phenomenon may have been apparent if we would have tested the *Akh*^1^ flies on diet with different sugar levels. Thus, it is important to take into account that in these kind of studies many factors may play a significant role, the ontogenic stage of experimental flies, the methods of gene manipulations selected, the conditions under which the experiments were conducted etc.

Taken together, our results conclusively demonstrate that AKH does impact phyiosiological and behavioral characteristics of senescence during aging in a subtle manner and this is sexually dimorphic. Other studies clearly implicate that signaling pathways that are stress- and nutrient-responsive (such as IIS/ FoxO, TOR, and JNK) play an important role in governing homeostasis and longevity (Wang et al., 2014). Thus, future studies should focus on involving a stress paradigm and also target the interactions of signaling pathways involved in energy homeostasis during the process of aging.

## Data Availability

The datasets generated during and/or analyzed during the current study are available from the corresponding author on request.

## Author Contributions

AB and NK conceived and designed the experiments; provided the ideas and supervised the work; and wrote the paper. AB, AT, and MM performed the experiments. AB, AT, and NK analyzed the data. DK contributed to the content. All authors read and approved the final version of the manuscript.

## Conflict of Interest Statement

The authors declare that the research was conducted in the absence of any commercial or financial relationships that could be construed as a potential conflict of interest.
