# Supplementary material for: Disruption of Adipokinetic Hormone Mediated Energy Homeostasis Has Subtle Effects on Physiology, Behavior and Lipid Status During Aging in Drosophila
Source: Front Physiol. 2018 Jul 20;9:949. doi: 10.3389/fphys.2018.00949 (PMC6062650; doi:10.3389/fphys.2018.00949)

Supplemental Figure S1: (**A**) Structural and storage lipid levels in various genotypes used in the study. Since gender differences were not significant, the data were pooled. While structural lipids did not differ significantly between genotypes, some differences were recorded in storage lipids especially with flies expression *Akh* ectopically (*EE-Akh*). (**B**) Stacked bar graph with different lipid species. PE - Phosphatidylethanolamine (cephalin) LysoPE – Lysophosphatidylethanolamine, PC - Phosphatidylcholine (lecithin), LysoPC – Lysophosphatidylcholine, PS – Phosphatidylserine, PI – Phosphatidylinositol, PG – Phosphatidylglycerol, DG – diacylglycerols, TG – triacylglycerols. Data in both graphs are recalculated per internal standard and per mg tissue sample.

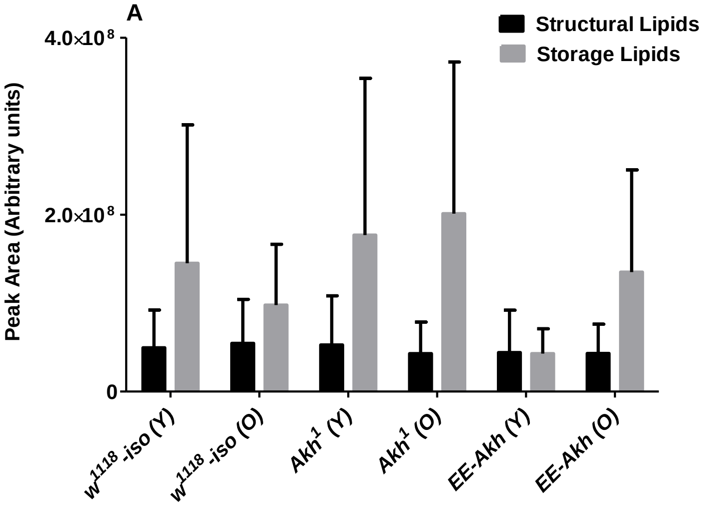

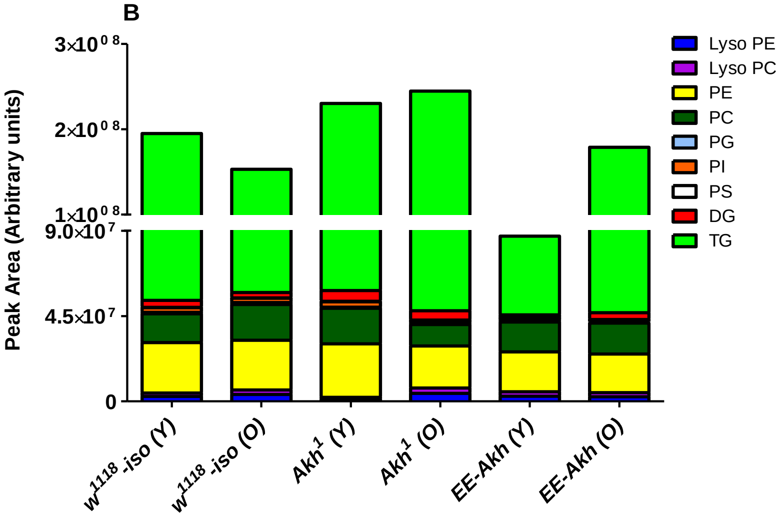

Supplement: Supplementary file 1 [file Image_1.PDF]
