# Supplementary material for: Disruption of Adipokinetic Hormone Mediated Energy Homeostasis Has Subtle Effects on Physiology, Behavior and Lipid Status During Aging in Drosophila
Source: Front Physiol. 2018 Jul 20;9:949. doi: 10.3389/fphys.2018.00949 (PMC6062650; doi:10.3389/fphys.2018.00949)

Supplemental Figure S2: (**A**) Structural and storage lipids in flies mutated in *Ak*h gene using CRISPR/Cas9. Since no differences were observed between males and females in distribution of structural or storage lipids the data for these lipid classes was pooled. No significant changes in structural lipids was observed between genotypes while some changes were observed in storage lipids with age. (**B**) Stacked bar graph with different lipid species. PE: Phosphatidylethanolamine (cephalin), LysoPE: Lysophosphatidylethanolamine, PC: Phosphatidylcholine (lecithin), LysoPC: Lysophosphatidylcholine, PS: Phosphatidylserine, PI: Phosphatidylinositol, PG: Phosphatidylglycerol, DG: diacylglycerols, TG: triacylglycerols. Data in both graphs are recalculated per internal standard and per mg tissue sample

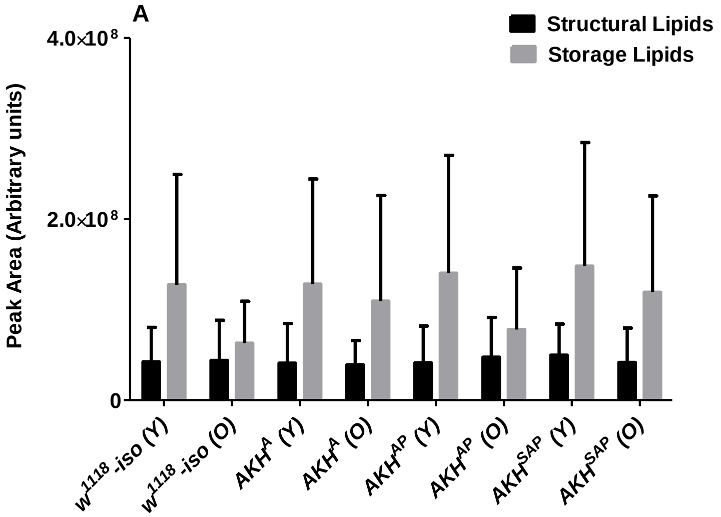

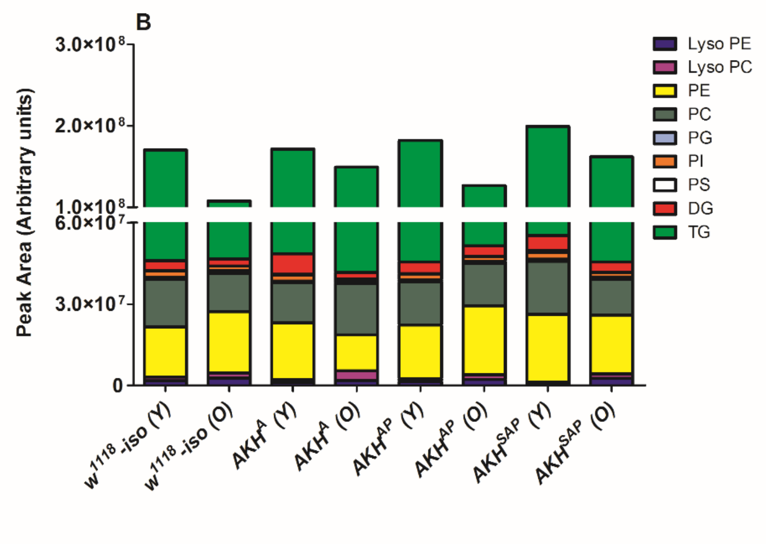

Supplement: Supplementary file 2 [file Image_2.PDF]
