# Supplementary material for: Disruption of Adipokinetic Hormone Mediated Energy Homeostasis Has Subtle Effects on Physiology, Behavior and Lipid Status During Aging in Drosophila
Source: Front Physiol. 2018 Jul 20;9:949. doi: 10.3389/fphys.2018.00949 (PMC6062650; doi:10.3389/fphys.2018.00949)

Supplemental Figure S3: RDA diagram of lipidomic data of three flies with mutated *Akh* gene using CRISPR/Cas9 and their isogenized control. Data was pooled between sexes since no sexually dimorphic distribution of lipid species was noted. This analysis was conducted irrespective of age. The multivariate analysis showed differences among depicted fly lines in p<0.01. The main lipid species responsible for data separation are depicted in the diagram.

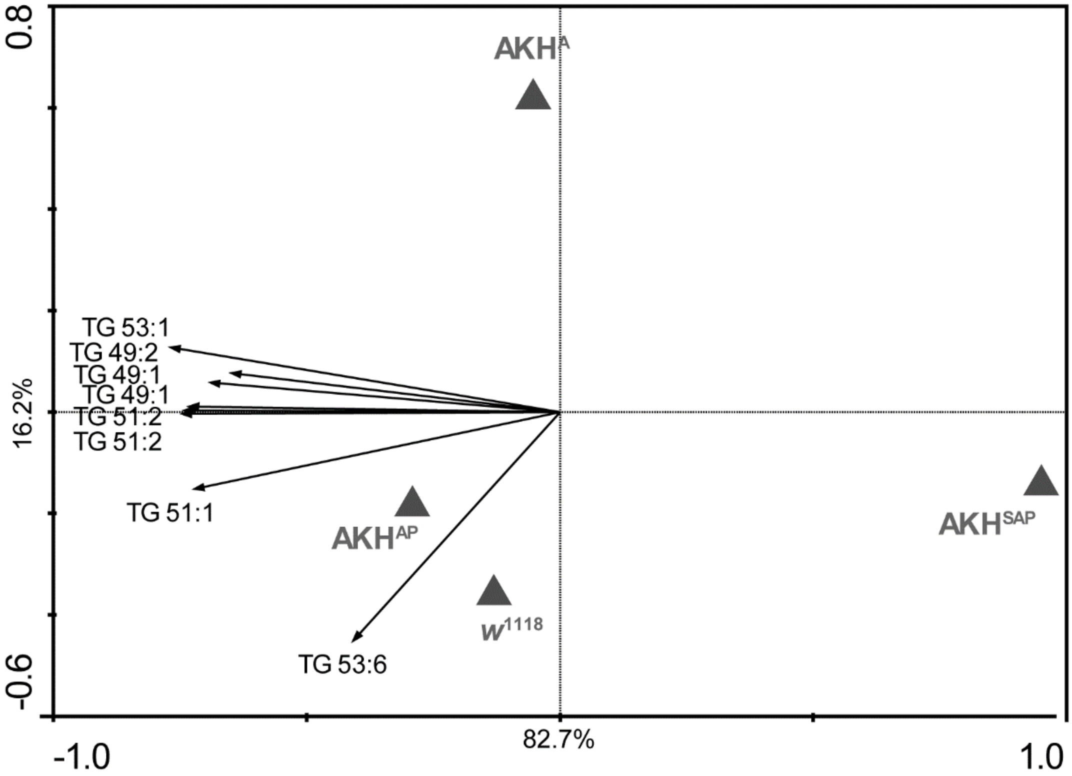

Supplement: Supplementary file 3 [file Image_3.PDF]
