# Supplementary material for: Disruption of Adipokinetic Hormone Mediated Energy Homeostasis Has Subtle Effects on Physiology, Behavior and Lipid Status During Aging in Drosophila
Source: Front Physiol. 2018 Jul 20;9:949. doi: 10.3389/fphys.2018.00949 (PMC6062650; doi:10.3389/fphys.2018.00949)

Supplemental Figure S4: RDA diagram of lipidomic data of three flies with mutated *Akh* gene using CRISPR/Cas9 and their isogenized control. Data was pooled between sexes since no sexually dimorphic distribution of lipid species was noted. Age was a factor that was taken into consideration in this analysis. The multivariate analysis showed differences among depicted fly p<0.01 The main responsible lipid species for data separation are depicted in the diagram.

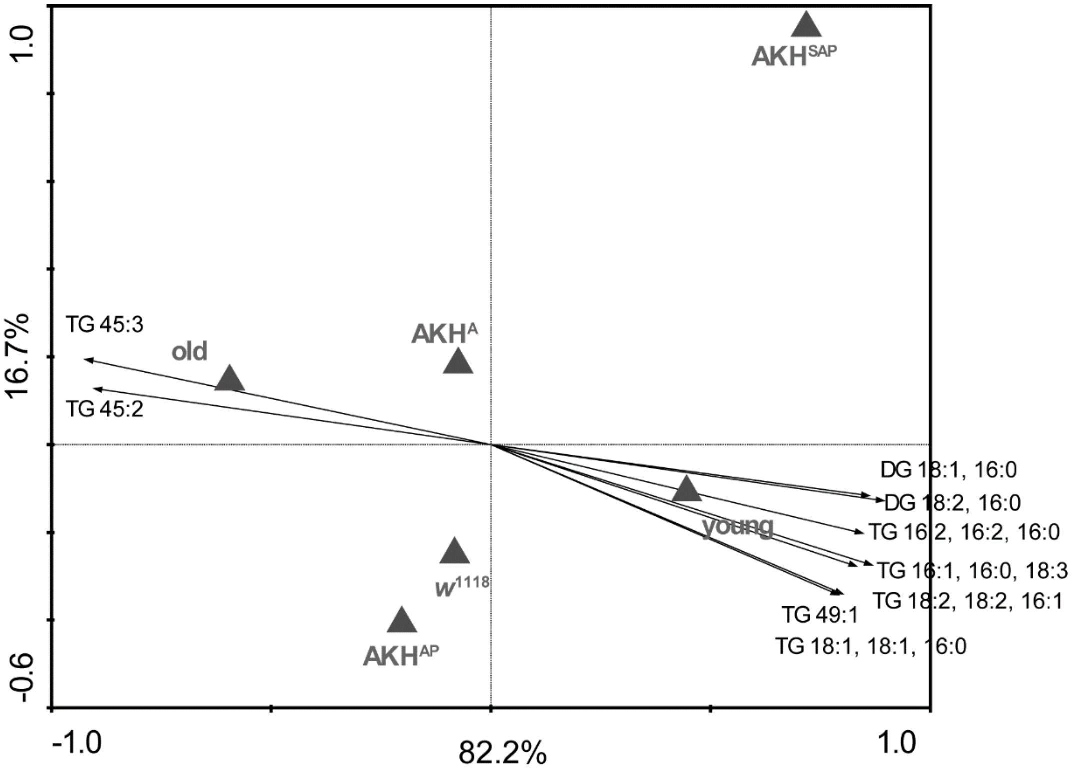

Supplement: Supplementary file 4 [file Image_4.PDF]
