# Supplementary material for: Disruption of Adipokinetic Hormone Mediated Energy Homeostasis Has Subtle Effects on Physiology, Behavior and Lipid Status During Aging in Drosophila
Source: Front Physiol. 2018 Jul 20;9:949. doi: 10.3389/fphys.2018.00949 (PMC6062650; doi:10.3389/fphys.2018.00949)
Supplement: Supplementary file 5 [file Table_1.PDF]

Supplemental Table S1: Primer sequences of genes used for qRT-PCR in this study.

**Gene**	**Forward primer (5'-3')**	**Reverse primer (5'-3')**
rp49	CAG TCG GAT CGA TAT GCT AAG GTG	TAA CCG ATG TTG GGC ATC AGA TAC T
AMPK	CAT CCG CAC ATC ATC AAG TT	TTC TCT GGC TTC AGG TCT CG
dTor	CAG GTT ATC CCG CAG CTT ATT	GCG GGT GAT TCT TTC CTA TGT
Akt	GCA GAG AAA TTC AGC TGG CAG CAA	TGA GTC TGT TCC GTA AGC GCA TGA
dFoxO	CCG CCA GCT TGG AAG ATA ATA	CAC GGG AAA GTT CTC CAG ATT
