# Supplementary material for: Disruption of Adipokinetic Hormone Mediated Energy Homeostasis Has Subtle Effects on Physiology, Behavior and Lipid Status During Aging in Drosophila
Source: Front Physiol. 2018 Jul 20;9:949. doi: 10.3389/fphys.2018.00949 (PMC6062650; doi:10.3389/fphys.2018.00949)
Supplement: Supplementary file 6 [file Table_2.PDF]

Supplemental Table S2: Mortality parameters derived from fitted Gompertz-Makeham model and maximum likelihood estimates (MLE). Mortality μ_x is given as $\mu_x = ae^{bx} + c$, where *a* is the baseline mortality rate (intercept), *b* is the age-dependent increase in mortality (slope), and *c* is the age-independent mortality.

**Genotypes**	**Gompertz-Makeham parameters**			**Actual lifespan**	**Fitted lifespan**	**% Error in lifespan**
	***a* (intercept)**	***b* (slope)**	***c* (constant)**			
	**MLE value**	**MLE value**	**MLE value**			
**Males**						
w^{1118}	2.1 (10^{-4})	0.021	5.1 (10^{-5})	57.4	57.6	0.002
Akh^{1}	2.3 (10^{-3})	0.064	3.1(10^{-9})	59.2	59.0	0.01
EE-Akh	5.0 (10^{-5})	0.133	2.3 (10^{-3})	54.6	54.8	0.002
**Females**						
w^{1118}	1.8 (10^{-3})	0.052	2.1 (10^{-9})	62.8	61.8	0.03
Akh^{1}	2.3 (10^{-3})	0.072	2.1 (10^{-9})	60.1	59.9	0.01
EE-Akh	8.0 (10^{-5})	0.133	2.6 (10^{-3})	58.6	58.2	0.002
