# Supplementary material for: Disruption of Adipokinetic Hormone Mediated Energy Homeostasis Has Subtle Effects on Physiology, Behavior and Lipid Status During Aging in Drosophila
Source: Front Physiol. 2018 Jul 20;9:949. doi: 10.3389/fphys.2018.00949 (PMC6062650; doi:10.3389/fphys.2018.00949)
Supplement: Supplementary file 7 [file Table_3.PDF]

**Supplemental Table S3:** Data representing the percentage of rhythmic flies (based on number of rhythmic individuals out of total number of flies tested for each genotype and gender), the period of rhythm (in constant darkness – DD) and the number of individuals of a genotype tested. Flies of each sex (male or female) and age (young, middle or old) from each genotype were tested separately. Period of rhythm is represented as mean (hours) ± SD.

| Sex/Age | Genotype | % Rhythmic | Period of rhythm in DD (Hrs) | Sample size (n) |
|---|---|---|---|---|
| **Male (young) 3-5 days** | $w^{1118}$ | 100 | 23.7± 0.2 | 32 |
| | $Akh^{1}$ | 95 | 23.9 ± 0.1 | 48 |
| | ***EE-Akh*** | 75 | 24.0 ± 0.3 | 32 |
| **Male (middle) ~ 30 days** | $w^{1118}$ | 94 | 24.3 ± 0.7 | 48 |
| | $Akh^{1}$ | 93 | 24.3 ± 0.6 | 48 |
| | ***EE-Akh*** | 44 | 24.3 ± 0.4 | 32 |
| **Male (old) ~ 45 days** | $w^{1118}$ | 20 | 24.3 ± 0.2 | 32 |
| | $Akh^{1}$ | 24 | 24.4 ± 0.2 | 38 |
| | ***EE-Akh*** | 31 | 24.5 ± 0.3 | 32 |
| **Female (young) 3-5 days** | $w^{1118}$ | 96 | 23.7 ± 0.5 | 36 |
| | $Akh^{1}$ | 98 | 23.4 ± 0.2 | 38 |
| | ***EE-Akh*** | 72 | 24.1 ± 0.5 | 42 |
| **Female (middle) ~ 30 days** | $w^{1118}$ | 74 | 24.6 ± 0.4 | 32 |
| | $Akh^{1}$ | 94 | 24.6 ± 0.6 | 42 |
| | ***EE-Akh*** | 65 | 24.2 ± 0.3 | 38 |
| **Female (old) ~ 45 days** | $w^{1118}$ | 18 | 26.3 ± 5.3 | 36 |
| | $Akh^{1}$ | 0 | Arrhythmic | 32 |
| | ***EE-Akh*** | 17 | 24.5 ± 6.0 | 32 |
