# Supplementary material for: Disruption of Adipokinetic Hormone Mediated Energy Homeostasis Has Subtle Effects on Physiology, Behavior and Lipid Status During Aging in Drosophila
Source: Front Physiol. 2018 Jul 20;9:949. doi: 10.3389/fphys.2018.00949 (PMC6062650; doi:10.3389/fphys.2018.00949)
Supplement: Supplementary file 8 [file Table_4.PDF]

Supplemental Table S4: Overall Representation of Lipid Classes found in all tested genotypes.

DG: Diacylglycerols, TG: Triacylglycerols, LysoPC: Lyso phosphatidylcholine, LysoPE: Lyso phosphatidylethanolamine, PC: Phosphatidylcholine, PE: Phosphatidylethanolamine, PI: Phosphatidylinositol, PG: Phosphatidylglycerol, PS: Phosphatidylcserine; m/z: mass to charge, ESI +: electrospray ionization technique in positive mode, rt: retention time, IDE: identification, CN:DB: Carbon Number: Double Bonds, ,: means the *sn* position is unknown, /: means the *sn* poition is known - *sn*-1/*sn*-2.

m/z	rt	IDE	
ESI +		lipid class	CN:DB or *sn*-1/*sn*-2
500	9.13	DG	26:1
502	10.95	DG	12:0,14:0
528	11.84	DG	28:1
530	13.43	DG	14:0, 14:0
534	11.74	DG	29:5
546	15.23	DG	30:6
548	17.82	DG	30:5
556	13.87	DG	16:1, 14:0
558	16.10	DG	30:0
582	14.3	DG	16:1, 16:1
584	16.58	DG	32:1
586	19.41	DG	32:0
608	15.05	DG	34:3
608	15.67	DG	34:3
610	16.98	DG	18:1, 16:1
610	17.46	DG	34:2
612	19.87	DG	34:1
614	23.05	DG	34:0
624	16.6	DG	35:2
626	19.21	DG	35:1
626	19.41	DG	35:1
628	20.34	DG	35:0
634	26.46	DG	36:4
636	17.92	DG	36:3
638	20.3	DG	36:2

638	21.02	DG	36:2
640	23.43	DG	36:1
642	26.05	DG	36:0
468	2.46	LysoPC	14:0
494	2.76	LysoPC	16:1
496	3.38	LysoPC	16:0
518	2.56	LysoPC	18:3
520	2.99	LysoPC	18:2
522	3.56	LysoPC	18:1
452	2.03	LysoPE	16:1
454	2.46	LysoPE	16:0
478	2.22	LysoPE	18:2
480	2.56	LysoPE	18:1
482	3.25	LysoPE	18:0
704	11.04	PC	14:0/16:1
706	12.95	PC	30:0
710	8.48	PC	31:5
712	10.21	PC	31:4
730	11.46	PC	16:1/16:1
730	11.74	PC	18:2/14:0
732	13.40	PC	16:0/16:1
734	15.53	PC	16:0/16:0
738	10.47	PC	33:5
738	10.86	PC	33:5
752	14.53	PC	34:5
754	16.13	PC	34:4
756	12.15	PC	18:2/16:1
756	12.27	PC	18:3/16:0
758	13.82	PC	18:1/16:1
758	14.06	PC	18:2/16:0
760	15.84	PC	18:1/16:0
762	18.53	PC	34:0
764	11.16	PC	35:6
764	11.86	PC	35:6
766	12.68	PC	35:5
766	13.10	PC	35:5

768	14.65	PC	35:4
780	17.09	PC	36:5
782	12.84	PC	18:2/18:2
782	13.16	PC	36:4
784	14.41	PC	18:1/18:2, 18:2/18:1
784	14.53	PC	18:0, 18:3
786	16.28	PC	18:1/18:1
786	16.83	PC	18:0/18:2
788	18.98	PC	18:1, 18:0
688	8.53	PE	16:1, 16:1
690	10.21	PE	16:0, 16:1
704	13.84	PE	33:1
712	8.01	PE	34:4
714	9.13	PE	16:1, 18:2
714	9.63	PE	16:0, 18:3
716	10.54	PE	16:1, 18:1
716	10.86	PE	16:0, 18:2
718	12.32	PE	16:0, 18:1
728	13.20	PE	35:4
728	14.06	PE	17:0, 18:3
730	14.53	PE	17:0, 18:2
732	16.15	PE	17:0, 18:1
736	7.19	PE	36:6
738	8.64	PE	36:5
740	9.78	PE	18:2, 18:2
740	9.91	PE	36:4
742	11.18	PE	18:2, 18:1
742	11.86	PE	18:0, 18:3
744	12.68	PE	18:1, 18:1
744	13.09	PE	18:0, 18:2
746	14.64	PE	18:0, 18:1
756	12.69	PE	37:3
758	17.04	PE	37:2
772	14.88	PE	38:2
772	15.43	PE	38:2
774	17.24	PE	38:1

710	6.88	PG	30:1
738	8.80	PG	32:1
762	7.88	PG	34:3
764	9.17	PG	18:1, 16:1
764	9.48	PG	18:2, 16:0
766	10.77	PG	18:1, 16:0
768	11.69	PG	34:0
788	8.48	PG	36:4
790	9.78	PG	36:3
792	11.10	PG	36:2
772	6.30	PI	28:0
798	6.67	PI	30:1
824	7.03	PI	16:1/16:1
826	8.59	PI	16:0/16:1
850	7.63	PI	16:1/18:2
852	8.91	PI	16:1/18:1
852	9.14	PI	16:0/18:2
854	10.57	PI	16:0/18:1
874	7.13	PI	36:5
876	8.18	PI	36:4
876	8.38	PI	36:4
878	9.52	PI	18:1/18:2
880	10.86	PI	36:2
880	11.23	PI	36:2
882	12.71	PI	36:1
774	8.91	PS	35:2
782	6.75	PS	36:5
784	7.81	PS	36:4
786	9.11	PS	36:3
786	9.37	PS	36:3
656	23.86	TG	12:0, 12:0, 12:0
682	24.54	TG	16:1, 10:0, 12:0
682	24.96	TG	12:0, 12:0, 14:1
684	27.21	TG	12:0, 12:0, 14:0
698	29.14	TG	40:7
700	39.72	TG	40:6

708	25.22	TG	40:2
708	25.50	TG	40:2
710	27.78	TG	12:0, 12:0, 16:1
710	27.89	TG	14:0, 12:0, 14:1
710	28.48	TG	14:0, 14:0, 12:1
712	31.15	TG	14:0, 12:0, 14:0
726	33.51	TG	42:7
736	28.24	TG	16:1, 14:1, 12:0
736	29.22	TG	14:1, 14:0, 14:1
738	31.75	TG	14:0, 16:1, 12:0
738	32.66	TG	14:0, 14:0, 14:1
740	35.60	TG	14:0, 14:0, 14:0
740	35.98	TG	16:0, 12:0, 14:0
754	38.51	TG	44:7
762	29.22	TG	44:3
762	29.62	TG	44:3
764	32.42	TG	44:2
764	33.25	TG	44:2
766	36.49	TG	14:0, 14:0, 16:1 and 14:0, 18:1, 12:0
768	40.37	TG	14:0, 14:0, 16:0
778	34.66	TG	45:2
778	41.36	TG	45:2
780	39.03	TG	45:1 or 46:8
780	43.05	TG	45:1 or 46:8
782	31.75	TG	45:0 or 46:7
790	33.25	TG	16:1, 16:1, 14:1
790	33.51	TG	16:1, 18:2, 12:0
790	33.79	TG	16:2, 16:0, 14:1
792	37.06	TG	16:1, 14:0, 16:1
794	40.70	TG	16:0, 16:1, 14:0
796	42.41	TG	16:0, 16:0, 14:0
806	39.52	TG	16:1, 15:0, 16:1 and 16:1, 17:1, 14:0
806	43.09	TG	47:2
808	44.28	TG	47:1 or 48:8
808	44.54	TG	47:1 or 48:8

812	34.67	TG	15:1, 15:1, 18:4
816	34.64	TG	48:5
816	35.08	TG	48:5
818	37.79	TG	16:1, 16:1, 16:1
820	40.88	TG	16:1, 16:1, 16:0
820	41.03	TG	16:0, 18:2, 14:0
822	42.54	TG	16:0, 18:1, 14:0
824	43.90	TG	48:0 or 49:7
824	43.93	TG	48:0 or 49:7
832	39.96	TG	49:3
832	43.35	TG	49:3
834	41.95	TG	49:2 or 50:9
834	44.60	TG	49:2 or 50:9
836	43.29	TG	49:1 or 50:8
836	46.25	TG	49:1 or 50:8
838	40.66	TG	49:0 or 50:7
842	35.67	TG	50:6
842	35.85	TG	50:5
844	39.04	TG	16:1, 18:2, 16:1
844	39.87	TG	50:4
846	41.19	TG	16:1, 16:1, 18:1
846	41.36	TG	16:0, 18:2, 16:1
848	42.67	TG	18:1, 16:1, 16:0
848	43.71	TG	16:1, 18:0, 16:1
850	43.97	TG	16:0, 18:1, 16:0
850	44.03	TG	18:0, 18:1, 14:0
852	45.42	TG	51:7
860	42.13	TG	51:3
860	44.78	TG	51:3
862	43.44	TG	51:2 or 52:9
862	46.25	TG	51:2 or 52:9
862	46.40	TG	51:2 or 52:9
864	40.83	TG	51:1 or 52:8
864	48.19	TG	51:1 or 52:8
870	39.96	TG	52:5
870	40.32	TG	52:5

872	41.67	TG	52:4
872	41.95	TG	52:4
874	42.82	TG	18:1, 18:1, 16:1
874	43.00	TG	18:1, 18:2, 16:0
874	49.55	TG	52:3
876	44.14	TG	18:1, 16:0, 18:1
876	44.20	TG	18:1, 18:0, 16:1
878	45.68	TG	18:0, 18:1, 16:0 and 20:0, 18:1, 14:0
880	41.16	TG	52:0 or 53:7
880	47.26	TG	52:0 or 53:7
890	48.38	TG	53:2 or 54:9
892	42.59	TG	53:1 or 54:8
892	50.36	TG	53:1 or 54:8
896	40.61	TG	53:0 or 54:6
898	42.13	TG	54:5
898	42.37	TG	54:5
900	43.15	TG	18:1, 18:1, 18:2
900	43.46	TG	18:2, 18:0, 18:2
902	44.28	TG	18:1, 18:1, 18:1
902	44.54	TG	54:3
904	45.72	TG	18:1, 18:0, 18:1
904	46.02	TG	18:1, 20:0, 16:1
906	47.36	TG	54:1
918	46.55	TG	55:2 or 56:9
932	47.36	TG	56:2
932	47.48	TG	56:2
934	49.53	TG	56:1 or 57:8
958	47.72	TG	58:3
960	49.41	TG	58:2
